# Supplementary material for: Detecting coalitions by optimally partitioning signed networks of political collaboration
Source: Sci Rep. 2020 Jan 30;10:1506. doi: 10.1038/s41598-020-58471-z (PMC6992702; doi:10.1038/s41598-020-58471-z)
Supplement: Supplementary file 1 — Movie S1. [file 41598_2020_58471_MOESM1_ESM.pdf]

Detecting coalitions by optimally partitioning signed networks of political collaboration

Samin Aref[1,2*] **and Zachary Neal**[3]

[1]Laboratory of Digital and Computational Demography, Max Planck Institute for Demographic Research, 18057 Rostock, Germany
[2]School of Computer Science, University of Auckland, 1142 Auckland, New Zealand
[3]Department of Psychology, Michigan State University, East Lansing, MI 48824, USA
[*]sare618@aucklanduni.ac.nz

This document describes all materials and methods for the article "Detecting coalitions by optimally partitioning signed networks of political collaboration" by Samin Aref and Zachary Neal.

This PDF file includes:

Supplementary Text
Figs. S1 to S4
Tables S1 to S4
Captions for Movies S1 to S2
Captions for Databases S1 to S2
References *(53-58)*

Other Supplementary Materials for this manuscript include the following:

Movies S1 to S2
Databases S1 to S2

Supplementary Text

Data availability

All network data and numerical results related to this study are publicly available with links provided in this document. The code for the optimization models used is this study is publicly available on a Github repository.

Analyzing networks of legislators

Most research on performance of political systems, and on the link between polarization and legislative effectiveness, has focused on legislators' ideological positions[53], role of political parties[54,55], and majority party size[41] within legislative chambers. However, others have suggested that a focus on parties to explain the dynamics of the US Congress is misguided[42]. Parties are administrative conveniences that facilitate coordination and often serve as a useful heuristic for their members' ideology, but because party affiliation is different from ideological position, a focus on political parties oversimplifies matters by assuming within-party ideological homogeneity and between-party ideological heterogeneity. Therefore, in this paper, we adopt a different approach, focusing more on networks of collaborations between legislators during a two-year session and less on legislators' political party affiliations. We find that this approach – examining polarization from the perspective of networks and structural balance – offers a better explanation of legislative effectiveness than political parties.

Our method of analysis is different from the conventional methods of indexing legislators partisanship[56–58] which place each legislator on a scale of liberal to conservative. These methods are shown to produce results correlating with important historical events in the US politics and therefore are standard practice in quantifying polarization[45]. While these methods indicate the political climate as a whole, they are not designed to take network relations of legislators into account.

Statistical analysis

The models shown in Fig. 5 (of the article) and discussed in the "Mediation in bill passage" section were estimated using Stata SE 13.1. All models were estimated separately for the US House of Representative and US Senate, and only standardized coefficients are reported. Model A was estimated as an ordinary bivariate linear regression using the following code in *Stata* software (release 13).

```
reg rate session, beta
```

Models B and C were estimated as structural equation models with maximum likelihood estimation using the following codes.

```
sem (party <- session) (rate <- party session)
sem (coalition <- session) (rate <- coalition session)
```

This estimation approach allows us to explicitly estimate the total indirect effect of time mediated by party control (in model B) or coalition partisanship (model C), and thus to test whether these variables help explain observed declines in bill passage rates.

Solving the continuous and discrete optimization models

The proposed continuous optimization model can be solved by any mathematical programming solver which supports linear programming (LP) models. In the Github repository, we explain using Gurobi solver (version 8.0) for solving the proposed LP model. The proposed discrete optimization model can be solved by any mathematical programming solver which supports 0/1 linear programming (binary linear) models. In the Github repository, we share our code for using Gurobi solver (version 8.0) to solve the proposed binary linear model.

The code for both the continuous and the discrete optimization models is available on a Github repository at https://github.com/saref/frustration-index-dense.

Using Gurobi for solving mathematical programming models

Our proposed algorithms are developed in Python 3.7 based on the mathematical programming models in[19,22] for computing the frustration index.

These optimization algorithms are distributed under an Attribution-NonCommercial-ShareAlike 4.0 International (CC BY-NC-SA 4.0) license. This means that one can use these algorithms for non-commercial purposes provided that they provide proper attribution for them by citing[19,22] and the current article. Copies or adaptations of the algorithms should be released under the similar license.

The following steps outline the process for academics to install the required software (*Gurobi* solver[32]) on your computer to be able to run the optimization algorithms:

1. Download and install Anaconda (Python 3.7 version) which allows you to run a Jupyter code. It can be downloaded from `https://www.anaconda.com/distribution/`. Note that you must select your operating system first and download the corresponding installer.

2. Register for an account on `gurobi.com/registration-general-reg/` to get a free academic license for using Gurobi. Note that Gurobi is a commercial software, but it can be registered with a free academic license if the user is affiliated with a recognized degree-granting academic institution. This involves creating an account on Gurobi website to be able to request a free academic license in step 5.

3. Download and install Gurobi Optimizer (versions 8.0 and above are recommended) which can be downloaded from `https://www.gurobi.com/downloads/gurobi-optimizer-eula/` after reading and agreeing to Gurobi's End User License Agreement.

4. Install Gurobi into Anaconda. You do this by first adding the Gurobi channel to your Anaconda channels and then installing the Gurobi package from this channel.

   From a terminal window issue the following command to add the Gurobi channel to your default search list

   ```
   conda config --add channels http://conda.anaconda.org/gurobi
   ```

   Now issue the following command to install the Gurobi package

   ```
   conda install gurobi
   ```

5. Request an academic license from `gurobi.com/downloads/end-user-license-agreement-academic/` and install the license on your computer following the instructions given on Gurobi license page.

   Completing these steps is explained in the following links (for version 8.1):

   for Windows `https://www.gurobi.com/documentation/8.1/quickstart_windows/installing_the_anaconda_py.html`,

   for Linux `gurobi.com/documentation/8.1/quickstart_linux/installing_the_anaconda_py.html`, and

   for Mac `gurobi.com/documentation/8.1/quickstart_mac/installing_the_anaconda_py.html`.

   After following the instructions above, open Jupyter Notebook which takes you to an environment (a new tab on your browser pops up on your screen) where you can open the main code (which is a file with .ipynb extension).

Visualization of opposing coalitions in Senate networks (Figures S1 to S3)

Figs. S1–S3 show the opposing coalitions in three selected Senate networks. Green and orange edges represent significantly many and significantly few co-sponsorships respectively. Blue and red nodes represent Democratic- and Republican-affiliated legislators respectively and nodes for independent legislators are shown in gray.

In Fig. S1, the network has a low level of balance $F(G) = 0.524$ (low polarization) and the larger coalition is relatively heterogeneous, which is reflected in the relatively small value of coalition control (0.559). In Fig. S2, the network has a low level of balance $F(G) = 0.603$ (low polarization) and the larger coalition is relatively homogeneous, which is reflected in the relatively high value of coalition control (0.911). In Fig. S3, the network has a high level of balance $F(G) = 0.953$ (high polarization) and the larger coalition is homogeneous (coalition control equals 0.981). The level of balance (coalition control) can also be observed from the colors of the edges (nodes) in Figs. S1–S3.

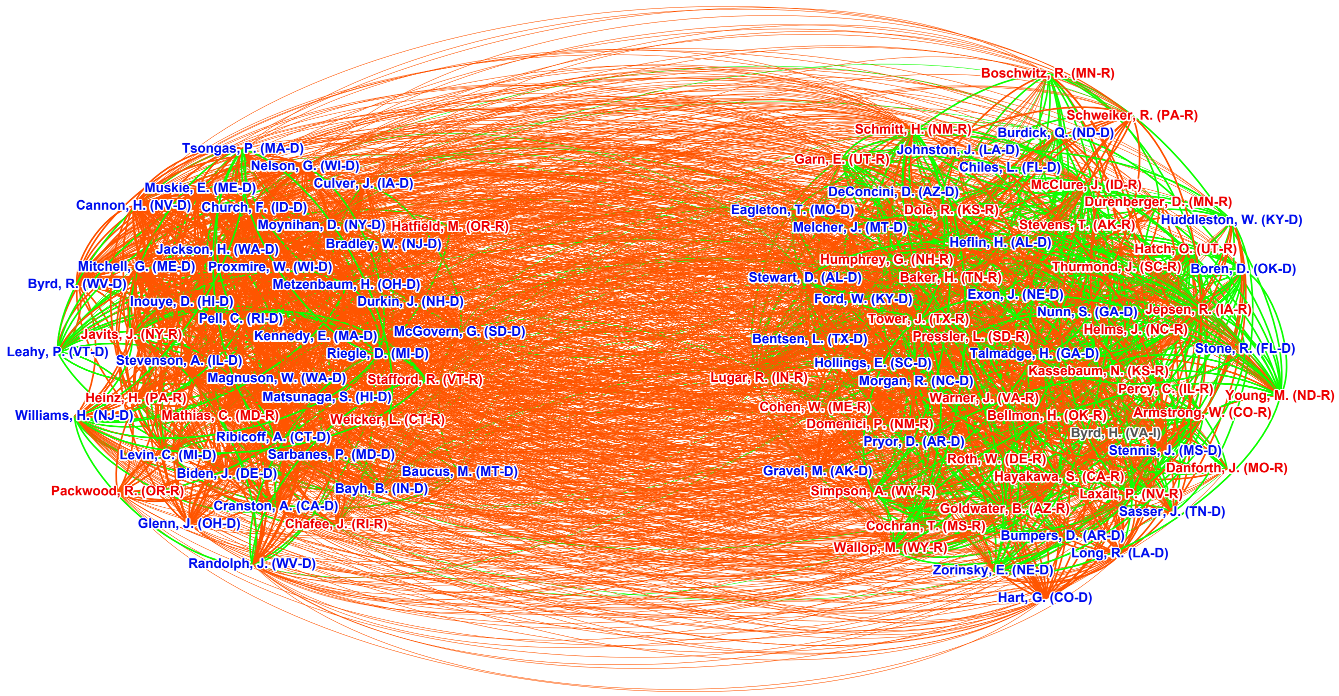

Figure S1. Opposing coalitions in the 96th session of the US Senate (1979). This network shows low balance and low coalition control.

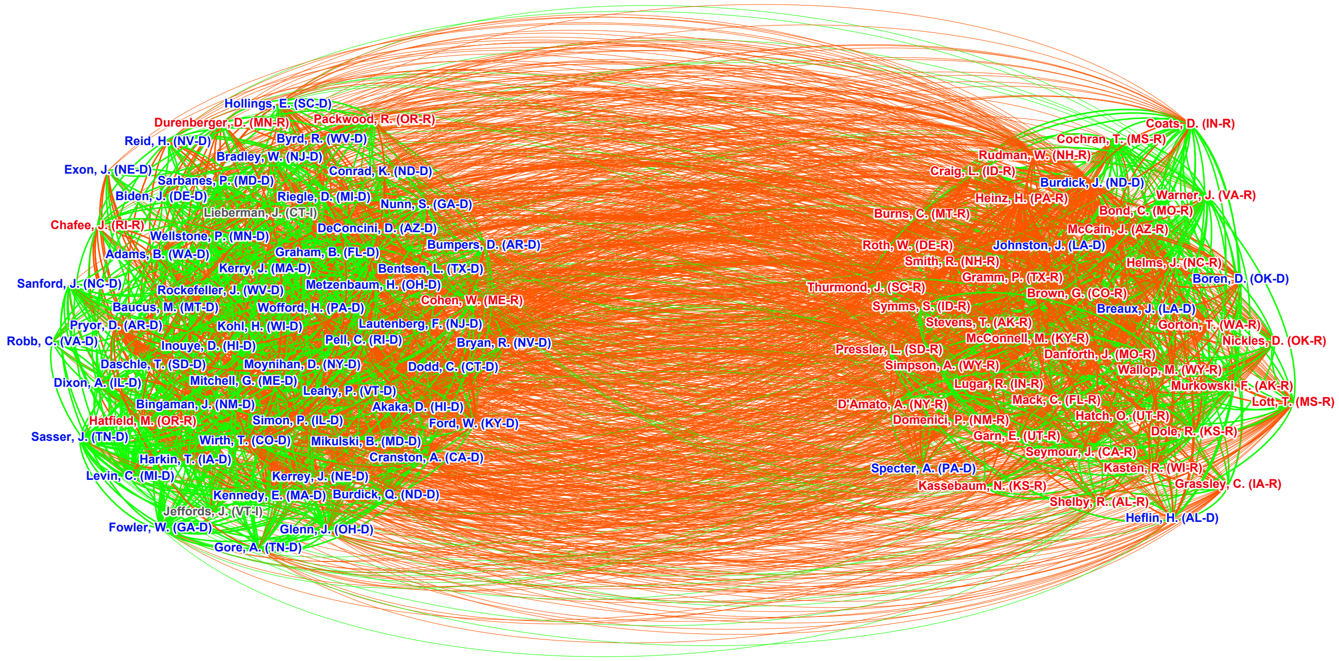

Figure S2. Opposing coalitions in the 102th session of the US Senate (1991). This network shows low balance and high coalition control.

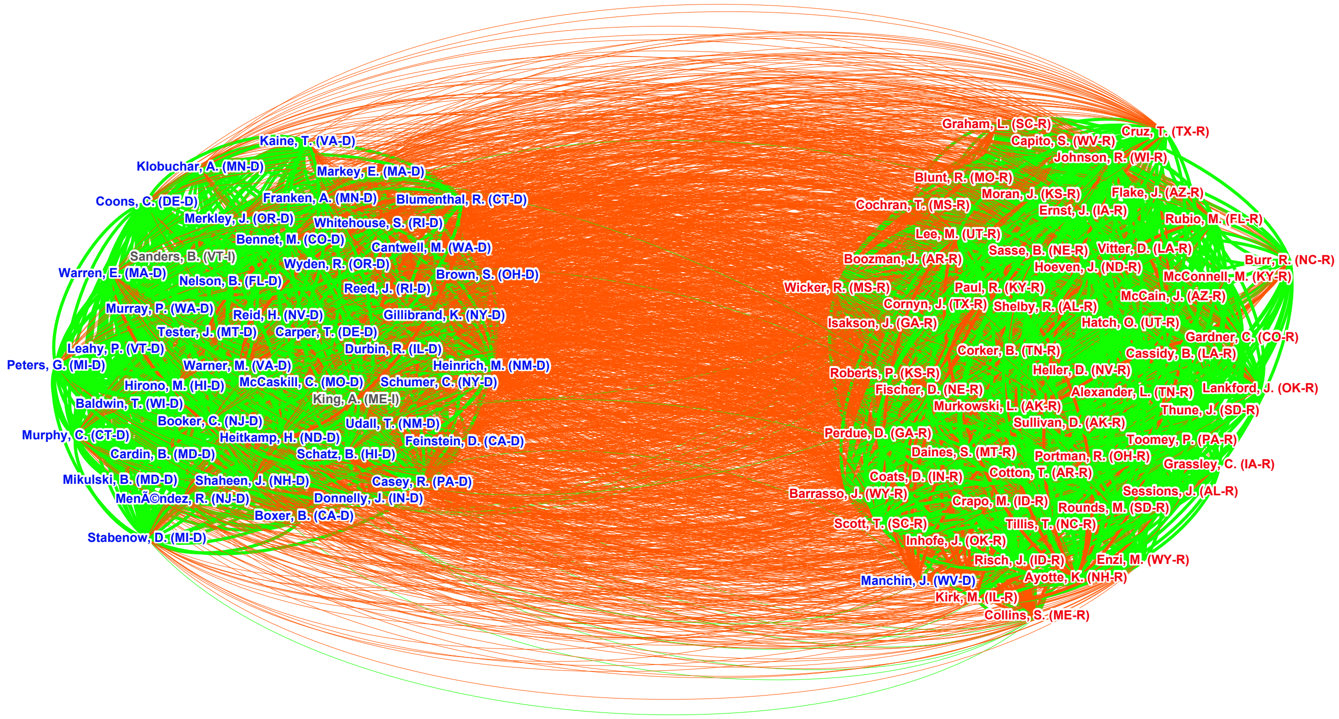

Figure S3. Opposing coalitions in the 114th session of the US Senate (2015). This network shows high balance and high coalition control.

Size of controlling coalitions

Using the optimal values of the x_i variables obtained by solving the discrete optimization model, we partition nodes of each network into two groups (subsets X^*, $V \setminus X^*$), namely nodes associated with x_i variables taking value 0 in the optimal solution and nodes whose corresponding variables take value 1 in the optimal solution.

For each signed network, either X^* or $V \setminus X^*$ has the larger set cardinality and therefore represents the largest coalition for the corresponding session. Fig. S4

shows the size of the largest and therefore controlling coalitions (winning coalitions[13]) in each signed network alongside the number of Democrats and Republicans in each session for both chambers.

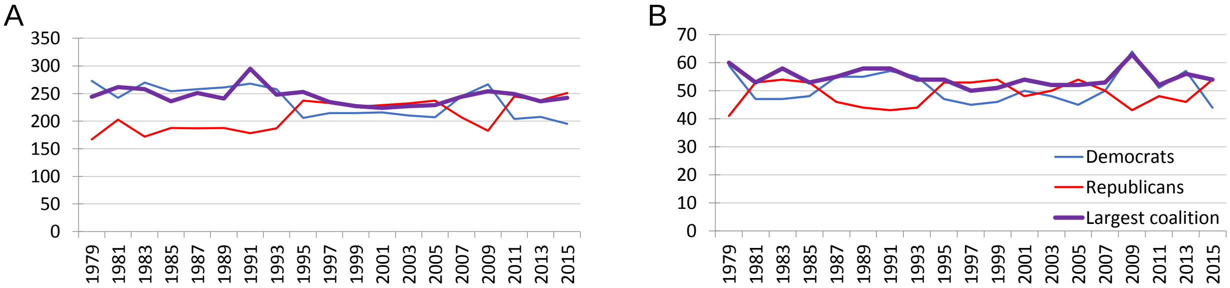

Figure S4. The number of legislators from the two main parties and the size of the largest coalition in (A) US House of Representatives and (B) US Senate over the time period 1979-2016

The controlling coalitions that our partitioning algorithm produces seem to be consistent with Riker's theory of *minimum winning coalitions*[13] which argues that politicians try to form winning (larger than majority limit) coalitions which are cohesive and minimal in size.

Legislative effectiveness

We compute our central outcome, legislative effectiveness, as the fraction of bills introduced in a chamber that are eventually signed into law by the president. Tables S2 and S4 report the number of bills introduced in the respective chamber and the number of these bills signed into law, as well as the passage rate, which measures legislative effectiveness. As the values in Tables S2 and S4 illustrate, the passage rate has generally declined over time. Importantly, this is not simply due to the introduction of more frivolous bills and thus is not a simple function of an increase in the denominator (i.e. bills introduced), which has fluctuated around a mean of 3386 in the Senate and 6483 in the House.

Additional numerical results for the Senate and House networks (Tables S1 to S4)

Table S1. Detailed properties and results for Senate networks

Session	Year	n	m	density	m^-	m^+	$T(G)$	$F(G)$	$L(G)$	Y^*
96	1979	101	2275	0.450	1870	405	0.410	0.524	541	541
97	1981	101	2073	0.410	1639	434	0.432	0.580	435	435
98	1983	101	2194	0.434	1676	518	0.511	0.521	525	525
99	1985	101	2177	0.431	1642	535	0.566	0.586	451	451
100	1987	101	2143	0.424	1535	608	0.594	0.525	509	509
101	1989	101	2445	0.484	1666	779	0.606	0.551	549	549
102	1991	102	2479	0.481	1768	711	0.635	0.603	492	492
103	1993	101	2257	0.447	1633	624	0.728	0.691	349	349
104	1995	102	2324	0.451	1715	609	0.777	0.811	220	220
105	1997	100	3002	0.606	2112	890	0.821	0.839	241	241
106	1999	102	2930	0.569	2108	822	0.852	0.816	269	269
107	2001	101	2522	0.499	1844	678	0.859	0.735	334	334
108	2003	100	2387	0.482	1759	628	0.862	0.828	205	205
109	2005	101	2823	0.559	2048	775	0.848	0.834	235	235
110	2007	102	2779	0.540	1934	845	0.853	0.851	207	207
111	2009	109	3645	0.619	2692	953	0.806	0.710	528	528
112	2011	101	3914	0.775	2693	1221	0.847	0.786	418	418
113	2013	105	3932	0.720	2554	1378	0.890	0.877	241	241
114	2015	100	3696	0.747	2261	1435	0.884	0.953	86	86

Table S2. Democrats (Dems), Republicans (Reps), controlling coalitions (CC), and bill passage in the Senate

Session	Dems	Reps	Size of CC	Dems in CC	Reps in CC	Party control	Coalition control	Bills introduced	Signed into law	Passage rate
96	59	41	60	26	33	18	0.559	3480	257	0.074
97	47	53	53	39	14	6	0.736	3396	210	0.062
98	47	54	58	44	14	7	0.759	3455	283	0.082
99	48	53	53	41	12	5	0.774	3386	304	0.090
100	55	46	55	16	39	9	0.709	3319	299	0.090
101	55	44	58	18	40	11	0.690	3659	277	0.076
102	57	43	58	51	5	14	0.911	3736	198	0.053
103	55	44	54	49	3	11	0.942	2801	172	0.061
104	47	53	54	4	50	6	0.926	2264	81	0.036
105	45	53	50	0	50	8	1.000	2715	141	0.052
106	46	54	51	0	51	8	1.000	3343	194	0.058
107	50	48	54	49	3	2	0.942	3234	71	0.022
108	48	50	52	47	3	2	0.940	3077	148	0.048
109	45	54	52	1	51	9	0.981	4163	151	0.036
110	50	50	53	49	2	0	0.961	3787	142	0.037
111	64	43	63	59	2	21	0.967	4101	120	0.029
112	51	48	52	49	1	3	0.980	3767	79	0.021
113	57	46	56	54	0	11	1.000	3067	77	0.025
114	44	54	54	1	53	10	0.981	3589	108	0.030

Table S3. Detailed properties and results for House networks

Session	Year	n	m	density	m^-	m^+	$T(G)$	$F(G)$	$L(G)$	Y^*
96	1979	442	51081	0.524	43097	7984	0.410	0.536	11845	11845
97	1981	447	49364	0.495	40304	9060	0.432	0.584	10260	10259
98	1983	444	48721	0.495	36592	12129	0.511	0.569	10494	10494
99	1985	443	49764	0.508	35716	14048	0.566	0.522	11885	11884
100	1987	446	50688	0.511	36780	13908	0.594	0.567	10979	10979
101	1989	449	56231	0.559	39394	16837	0.606	0.565	12232	12231
102	1991	447	58067	0.583	39726	18341	0.635	0.590	11914	11914
103	1993	446	59092	0.595	40290	18802	0.728	0.688	9222	9222
104	1995	445	62154	0.629	44537	17617	0.777	0.797	6299	6299
105	1997	449	66701	0.663	46121	20580	0.821	0.813	6238	6238
106	1999	442	63652	0.653	42753	20899	0.852	0.830	5395	5395
107	2001	447	63851	0.641	43246	20605	0.859	0.848	4866	4866
108	2003	444	66277	0.674	44397	21880	0.862	0.847	5057	5057
109	2005	445	66700	0.675	45420	21280	0.848	0.829	5695	5695
110	2007	452	70923	0.696	47412	23511	0.853	0.842	5618	5618
111	2009	451	70160	0.691	47656	22504	0.806	0.775	7877	7877
112	2011	450	77872	0.771	51084	26788	0.847	0.844	6063	6063
113	2013	447	75771	0.760	48226	27545	0.890	0.880	4533	4533
114	2015	446	75180	0.758	48602	26578	0.884	0.872	4801	4801

Table S4. Democrats (Dems), Republicans (Reps), controlling coalitions (CC), and bill passage in the House

Session	Dems	Reps	Size of CC	Dems in CC	Reps in CC	Party control	Coalition control	Bills introduced	Signed into law	Passage rate
96	273	167	244	90	154	106	0.631	9101	477	0.052
97	242	203	262	77	185	39	0.706	8093	317	0.039
98	270	172	258	93	165	98	0.640	7105	392	0.055
99	254	188	236	218	17	66	0.928	6499	381	0.059
100	258	187	251	228	22	71	0.912	6263	458	0.073
101	261	188	241	227	14	73	0.942	6664	387	0.058
102	268	178	295	241	53	90	0.820	6775	411	0.061
103	258	187	248	239	8	71	0.968	5739	301	0.052
104	206	237	253	23	230	31	0.909	4542	253	0.056
105	215	233	235	16	219	18	0.932	5014	262	0.052
106	215	226	227	206	20	11	0.912	5815	410	0.071
107	216	229	224	5	219	13	0.978	5890	312	0.053
108	210	232	227	4	223	22	0.982	5546	356	0.064
109	207	237	229	2	227	30	0.991	6538	332	0.051
110	245	207	244	238	6	38	0.975	7441	314	0.042
111	267	183	254	252	1	84	0.996	6669	265	0.040
112	204	245	249	9	240	41	0.964	6845	205	0.030
113	208	238	236	5	231	30	0.979	6016	219	0.036
114	195	251	242	1	241	56	0.996	6634	218	0.033

Movie: Animated versions of coalitions in networks

Animated versions of the opposing coalitions of the networks is available online at https://saref.github.io/SI/AN2019/Senate_coalitions.mp4 for the Senate and at https://saref.github.io/SI/AN2019/House_coalitions.mp4 for the House of Representatives. Looking at the colors of edges we observe that over time, more edges within (between) groups become green (orange) which shows that the networks become more partially balanced and therefore more polarized. If we look at the colors of the nodes (which represent party affiliations), we see that the coalitions become more homogeneous (partisan) over time. These two changes show the two aspects of increase in partisan polarization over time.

Dataset: a-sign-of-the-times.xlsx

All 38 signed networks used in this study are available as adjacency matrices stored in an Excel file accessible in a public *Figshare* data repository[4]. These data are distributed under a CC-BY 4.0 license. This means that one can use these data provided that they provide proper attribution for them by citing the two articles[5,6].

Each tab (sheet) contains the signed network for a chamber of congress indexed using H for House, S for Senate and a number for session (e.g. S98 means the 98th session of the Senate). The first row identifies the representative or senator, with their party affiliation and state; these are square matrices with columns arranged in the same order. In each matrix, a value of 1 (-1) means that the congresspeople associated with that matrix entry have a positive (negative) tie in the network associated with that chamber and session. Likewise, a value of 0 means that the reciprocal congresspeople have no relationship in that network. Relationships are inferred from bill co-sponsorship data using the Stochastic Degree Sequence Model (SDSM).

The 19 signed networks for the US Senate have slightly over 100 nodes, each representing a senator, and total edge count between 2073–3932, which results in density values between 0.41 and 0.78. The proportions of negative edges are within the range of 0.61 and 0.82. The 19 signed networks for the US House of Representatives have slightly over 435 nodes, each representing a representative, and a total edge count between 48721 and 77872, which results in densities in the range of 0.50–0.77. The proportion of negative edges varies between 0.64 and 0.84. The networks are slightly larger than the number of legislative seats in their respective chambers because a single seat may be occupied by more than one legislator during a single session, for example due to a death, retirement, or resignation. Accordingly, the nodes in these networks represent individual legislators, not legislative seats.

Dataset: {House/Senate}-coalition-membership-results.csv

The results on globally optimal solutions to the optimization model for computing the frustration index for House and Senate are available in comma-separated values format (two individual csv files) at https://saref.github.io/SI/AN2019/House_coalition_membership_results.csv and https://saref.github.io/SI/AN2019/Senate_coalition_membership_results.csv .

The first column contains node IDs and the first row contains session numbers. The entry at the intersection of row indexed r and column indexed c represents the optimal value of the x_i variable for a given node (node r) in a given session (session c).

Supplementary legends

Fig. S1: Opposing coalitions in the 96th session of the US Senate (1979). This network shows low balance and low coalition control.
Fig. S2: Opposing coalitions in the 102th session of the US Senate (1991). This network shows low balance and high coalition control.
Fig. S3: Opposing coalitions in the 114th session of the US Senate (2015). This network shows high balance and high coalition control.
Fig. S4: The number of legislators from the two main parties and the size of the largest coalition in (A) US House of Representatives and (B) US Senate over the time period 1979-2016
Table S1: Detailed properties and results for Senate networks
Table S2: Democrats (Dems), Republicans (Reps), controlling coalitions (CC), and bill passage in the Senate
Table S3: Detailed properties and results for House networks
Table S4: Democrats (Dems), Republicans (Reps), controlling coalitions (CC), and bill passage in the House

References

1. Ribeiro, H. V., Alves, L. G. A., Martins, A. F., Lenzi, E. K. & Perc, M. The dynamical structure of political corruption networks. *J. Complex Networks* **6**, 989–1003, DOI: 10.1093/comnet/cny002 (2018).
2. Colliri, T. & Zhao, L. Analyzing the bills-voting dynamics and predicting corruption-convictions among Brazilian congressmen through temporal networks. *Sci. Reports* **9**, 16754, DOI: 10.1038/s41598-019-53252-9 (2019).
3. Faustino, J., Barbosa, H., Ribeiro, E. & Menezes, R. A data-driven network approach for characterization of political parties' ideology dynamics. *Appl. Netw. Sci.* **4**, 48, DOI: 10.1007/s41109-019-0161-0 (2019).
4. Neal, Z. A Sign of the Times: Dataset of US Congress signed network backbones from co-sponsorship data, 1973-2016. *figshare* http://dx.doi.org/10.6084/m9.figshare.8096429 (2019).
5. Neal, Z. The backbone of bipartite projections: Inferring relationships from co-authorship, co-sponsorship, co-attendance and other co-behaviors. *Soc. Networks* **39**, 84–97, DOI: 10.1016/j.socnet.2014.06.001 (2014).
6. Neal, Z. A sign of the times? Weak and strong polarization in the U.S. Congress, 1973–2016. *Soc. Networks* **60**, 103 – 112, DOI: 10.1016/j.socnet.2018.07.007 (2020).
7. Terzi, E. & Winkler, M. A spectral algorithm for computing social balance. In Frieze, A., Horn, P. & Prałat, P. (eds.) *Proceedings of International Workshop on Algorithms and Models for the Web-Graph*, WAW 2011, 1–13, DOI: 10.1007/978-3-642-21286-4_1 (Springer, 2011).
8. Zaslavsky, T. Balanced decompositions of a signed graph. *J. Comb. Theory, Ser. B* **43**, 1–13, DOI: 10.1016/0095-8956(87)90026-8 (1987).
9. Facchetti, G., Iacono, G. & Altafini, C. Computing global structural balance in large-scale signed social networks. *Proc. Natl. Acad. Sci.* **108**, 20953–20958, DOI: 10.1073/pnas.1109521108 (2011).
10. Aref, S. & Wilson, M. C. Measuring partial balance in signed networks. *J. Complex Networks* **6**, 566–595, DOI: 10.1093/comnet/cnx044 (2018).
11. Harary, F. On the measurement of structural balance. *Behav. Sci.* **4**, 316–323, DOI: 10.1002/bs.3830040405 (1959).
12. Harary, F. & Kabell, J. A. A simple algorithm to detect balance in signed graphs. *Math. Soc. Sci.* **1**, 131–136, DOI: 10.1016/0165-4896(80)90010-4 (1980).
13. Riker, W. H. *The theory of political coalitions* (Yale University Press, 1962).
14. Hüffner, F., Betzler, N. & Niedermeier, R. Separator-based data reduction for signed graph balancing. *J. Comb. Optim.* **20**, 335–360, DOI: 10.1007/s10878-009-9212-2 (2010).
15. Gong, M., Cai, Q., Ma, L., Wang, S. & Lei, Y. Network structure balance analytics with evolutionary optimization. In *Computational Intelligence for Network Structure Analytics*, 135–199, DOI: 10.1007/978-981-10-4558-5_4 (Springer, 2017).
16. Traag, V. A., Doreian, P. & Mrvar, A. Partitioning signed networks. In Doreian, P., Batagelj, V. & Ferligoj, A. (eds.) *Advances in network clustering and blockmodeling*, chap. 8 (Wiley-Interscience, 2018).
17. Hua, J., Yu, J. & Yang, M.-S. Fast clustering for signed graphs based on random walk gap. *Soc. Networks* DOI: 10.1016/j.socnet.2018.08.008 (2018).
18. Brusco, M. J. & Doreian, P. Partitioning signed networks using relocation heuristics, tabu search, and variable neighborhood search. *Soc. Networks* **56**, 70–80, DOI: 10.1016/j.socnet.2018.08.007 (2019).
19. Aref, S., Mason, A. J. & Wilson, M. C. A modeling and computational study of the frustration index in signed networks. *Networks* **75**, 95–110, DOI: 10.1002/net.21907 (2020).
20. Heider, F. Social perception and phenomenal causality. *Psychol. Rev.* **51**, 358–378, DOI: 10.1037/h0055425 (1944).
21. Cartwright, D. & Harary, F. Structural balance: a generalization of Heider's theory. *Psychol. Rev.* **63**, 277–293, DOI: 10.1037/h0046049 (1956).
22. Aref, S., Mason, A. J. & Wilson, M. C. Computing the line index of balance using integer programming optimisation. In Goldengorin, B. (ed.) *Optimization Problems in Graph Theory*, 65–84, DOI: 10.1007/978-3-319-94830-0_3 (Springer, 2018).
23. Aref, S. & Wilson, M. C. Balance and frustration in signed networks. *J. Complex Networks* **7**, 163–189, DOI: 10.1093/comnet/cny015 (2019).
24. Harary, F. Graphing conflict in international relations. *The papers Peace Sci. Soc.* **27**, 1–10 (1977).

25. Abelson, R. P. & Rosenberg, M. J. Symbolic psycho-logic: A model of attitudinal cognition. *Behav. Sci.* **3**, 1–13, DOI: 10.1002/bs.3830030102 (1958).

26. Kernighan, B. W. & Lin, S. An efficient heuristic procedure for partitioning graphs. *Bell Syst. Tech. J.* **49**, 291–307, DOI: 10.1002/j.1538-7305.1970.tb01770.x (1970).

27. Girvan, M. & Newman, M. E. J. Community structure in social and biological networks. *Proc. Natl. Acad. Sci.* **99**, 7821–7826, DOI: 10.1073/pnas.122653799 (2002).

28. Clauset, A., Newman, M. E. J. & Moore, C. Finding community structure in very large networks. *Phys. Rev. E* **70**, 066111, DOI: 10.1103/PhysRevE.70.066111 (2004).

29. Raghavan, U. N., Albert, R. & Kumara, S. Near linear time algorithm to detect community structures in large-scale networks. *Phys. Rev. E* **76**, 036106, DOI: 10.1103/PhysRevE.76.036106 (2007).

30. Cordasco, G. & Gargano, L. Community detection via semi-synchronous label propagation algorithms. In *2010 IEEE International Workshop on: Business Applications of Social Network Analysis (BASNA)*, 1–8, DOI: 10.1109/BASNA.2010.5730298 (2010).

31. Parés, F. *et al.* Fluid communities: A competitive, scalable and diverse community detection algorithm. In Cherifi, C., Cherifi, H., Karsai, M. & Musolesi, M. (eds.) *Complex Networks & Their Applications VI*, 229–240, DOI: 10.1007/978-3-319-72150-7_19 (Springer International Publishing, Cham, 2018).

32. Gurobi Optimization Inc. Gurobi optimizer reference manual (2018). Url: www.gurobi.com/documentation/8.0/refman/index.html date accessed 1 June 2018.

33. Layman, G. C., Carsey, T. M. & Horowitz, J. M. Party polarization in american politics: Characteristics, causes, and consequences. *Annu. Rev. Polit. Sci.* **9**, 83–110, DOI: 10.1146/annurev.polisci.9.070204.105138 (2006).

34. Zhang, Y. *et al.* Community structure in congressional cosponsorship networks. *Phys. A* **387**, 1705–1712, DOI: 10.1016/j.physa.2007.11.004 (2008).

35. Waugh, A. S., Pei, L., Fowler, J. H., Mucha, P. J. & Porter, M. A. Party polarization in congress: A network science approach. *arXiv* (2011). :0907.3509 (25 Jul 2011).

36. Moody, J. & Mucha, P. J. Portrait of political party polarization. *Netw. Sci.* **1**, 119–121, DOI: 10.1017/nws.2012.3 (2013).

37. Olson, D. M. & Nonidez, C. T. Measures of legislative performance in the U.S. House of Representatives. *Midwest J. Polit. Sci.* **16**, 269–277, DOI: 10.2307/2110060 (1972).

38. Frantzich, S. Who makes our laws? The legislative effectiveness of members of the U.S. congress. *Legislative Stud. Q.* **4**, 409–428, DOI: 10.2307/439582 (1979).

39. Volden, C. & Wiseman, A. E. *Legislative effectiveness in the United States Congress: The lawmakers* (Cambridge university press, 2014).

40. Mayhew, D. R. *Divided we govern: Party control, lawmaking, and investigations, 1946-2002* (Yale university press, 2005).

41. Moore, D. W. Legislative effectiveness and majority party size: A test in the indiana house. *The J. Polit.* **31**, 1063–1079, DOI: 10.2307/2128358 (1969).

42. Mayhew, D. R. *Congress: The Electoral Connection* (Yale university press, 1974).

43. Monroe, N. W., Roberts, J. M. & Rohde, D. W. *Why Not Parties? Party Effects in the United States Senate* (University of Chicago Press, 2008).

44. Fowler, J. H. Legislative cosponsorship networks in the US House and Senate. *Soc. Networks* **28**, 454–465, DOI: 10.1016/j.socnet.2005.11.003 (2006).

45. Andris, C. *et al.* The rise of partisanship and super-cooperators in the U.S. House of Representatives. *PloS one* **10**, 1–14, DOI: 10.1371/journal.pone.0123507 (2015).

46. Arinik, N., Figueiredo, R. & Labatut, V. Analysis of roll-calls in the European parliament by multiple partitioning of multiplex signed networks. *Soc. Networks (in press)* (2019). Doi: 10.1016/j.socnet.2019.02.001 (26 November 2018).

47. Porter, M. A., Mucha, P. J., Newman, M. E. J. & Warmbrand, C. M. A network analysis of committees in the U.S. House of Representatives. *Proc. Natl. Acad. Sci.* **102**, 7057–7062, DOI: 10.1073/pnas.0500191102 (2005).

48. Desmarais, B. A., Moscardelli, V. G., Schaffner, B. F. & Kowal, M. S. Measuring legislative collaboration: The Senate press events network. *Soc. Networks* **40**, 43–54, DOI: 10.1016/j.socnet.2014.07.006 (2015).

49. Serrano, M. Á., Boguñá, M. & Vespignani, A. Extracting the multiscale backbone of complex weighted networks. *Proc. Natl. Acad. Sci.* **106**, 6483–6488, DOI: 10.1073/pnas.0808904106 (2009).

50. Dianati, N. Unwinding the hairball graph: Pruning algorithms for weighted complex networks. *Phys. Rev. E* **93**, 012304, DOI: 10.1103/PhysRevE.93.012304 (2016).

51. Latapy, M., Magnien, C. & Vecchio, N. D. Basic notions for the analysis of large two-mode networks. *Soc. Networks* **30**, 31 – 48, DOI: 10.1016/j.socnet.2007.04.006 (2008).

52. Thomas, S. & Grofman, B. The effects of congressional rules about bill cosponsorship on duplicate bills: changing incentives for credit claiming. *Public Choice* **75**, 93–98, DOI: 10.1007/BF01053883 (1993).

53. Olzak, S., Soule, S. A., Coddou, M. & Muñoz, J. Friends or foes? How social movement allies affect the passage of legislation in the U.S. Congress. *Mobilization: An Int. Q.* **21**, 213–230, DOI: 10.17813/1086-671X-21-2-213 (2016). https://doi.org/10.17813/1086-671X-21-2-213.

54. Anderson, W. D., Box-Steffensmeier, J. M. & Sinclair-Chapman, V. The keys to legislative success in the U.S. House of Representatives. *Legislative Stud. Q.* **28**, 357–386, DOI: 10.3162/036298003X200926 (2003).

55. Finocchiaro, C. J. & Rohde, D. W. War for the floor: Partisan theory and agenda control in the U.S. House of Representatives. *Legislative Stud. Q.* **33**, 35–61, DOI: 10.3162/036298008783743273 (2008).

56. Poole, K. T. & Rosenthal, H. The polarization of american politics. *The J. Polit.* **46**, 1061–1079, DOI: 10.2307/2131242 (1984).

57. Poole, K. T. & Rosenthal, H. *Congress: A political-economic history of roll call voting* (Oxford University Press on Demand, 2000).

58. Cox, G. W. & Poole, K. T. On measuring partisanship in roll-call voting: The US House of Representatives, 1877-1999. *Am. J. Polit. Sci.* 477–489, DOI: 10.2307/3088393 (2002).
